# Liaison Psychiatry During the Peak of the Coronavirus Pandemic: A Description of Referrals and Interventions

**DOI:** 10.3389/fpsyt.2021.555080

**Published:** 2021-12-09

**Authors:** Mayte López-Atanes, Juan Pablo González-Briceño, Adrián Abeal-Adham, Sara Fuertes-Soriano, Janire Cabezas-Garduño, Álvar Peña-Rotella, Margarita Sáenz-Herrero

**Affiliations:** ^1^Department of Psychiatry, Cruces University Hospital, Osakidetza-Basque Health Service, Barakaldo, Spain; ^2^Department of Preventive Medicine and Public Health, Cruces University Hospital, Osakidetza-Basque Health Service, Barakaldo, Spain; ^3^Department of Psychiatry, Biocruces Bizkaia Health Research Institute, Cruces University Hospital, Barakaldo, Spain

**Keywords:** COVID-19, SARS-CoV-2, liaison psychiatry, mental health, psychological counseling

## Abstract

**Introduction:** The novel coronavirus SARS-CoV-2 belongs to the coronavirus family, a group of viruses that can cause upper respiratory infections in humans. Among other symptoms, it can present as an asymptomatic infection or as a more severe disease requiring hospitalization. Neuropsychiatric symptoms have been described in the acute phase of the illness and as long-term repercussions. We describe the characteristics and interventions in those COVID-19 patients referred to our liaison psychiatry service.

**Materials and Methods:** This is a cross-sectional descriptive study. This study was carried out within the Department of Psychiatry of Cruces University Hospital (Basque Country, Spain). Data from each psychiatric consultation within our consultation-liaison service were consecutively obtained for 1 month from March 17 to April 17, 2020. We recruited data regarding clinical and referral characteristics and psychiatric interventions.

**Results:** Of a total of 721 SARS-CoV-2 hospitalizations, 43 (5.6%) patients were referred to our psychiatry liaison service. The median age was 61 years old, and 62.8% were women. The infectious disease department was the most frequent petitioner (37.2%), and the most common reason for referral was patient anxiety (25.6%). A total of 67.4% of patients received psychological counseling and 55.8% received some pharmacological approach, with a median of 3.7 visits/calls per patient. In addition, 20.3% needed a medication switch due to potential interactions between psychotropics and drugs used to treat SARS-CoV-2.

**Discussion:** In our study, up to 5.6% of SARS-CoV-2 hospitalized patients needed a psychiatric evaluation, especially for anxiety and mood symptoms. Psychosocial factors associated with the pandemic, drugs used to treat the infection, or a direct causative effect of the virus may explain our findings.

## Introduction

SARS-CoV-2 is a novel coronavirus first detected in the Wuhan area, China, in December 2019 (1). It has progressively expanded through China and eventually became an international pandemic. Our study took place in the Cruces University hospital, a third-level hospital in Basque Country, Spain. At the moment of this study, in March 2020, our hospital admitted more than 700 inpatients due to SARS-CoV-2 infection, and Spain was, in fact, one of the more severely affected countries (2).

SARS-CoV-2 is a coronavirus (3), which is a group of single-stranded RNA viruses that can cause upper respiratory infections in humans. The COVID-19 symptom spectrum is vast and ranges from an asymptomatic infection in up to 20% of patients to severe pneumonia requiring hospitalization and death (1, 4, 5). Although it primarily affects the airway and the lungs, the virus can also attack the kidneys, the liver, and the central nervous system and cause multi-organ failure. In China, the mortality rate has been estimated to be 1.38%, increasing with age and increasing to 13.4% in those aged 80 or older (5).

Men have a higher mortality rate and an increased risk of admission to the ICU than women (6). Although the organic prognosis is worse, the psychological impact remains better in men: women infected with SARS-CoV-2 report more perceived helplessness than men do (7) and have higher scores in depression and anxiety scales (8). It has to be clarified whether biological or sociocultural variables explain these gender differences.

### Neuropsychiatric Manifestations of the Infection

Like other viruses of this group, the SARS-CoV-2 has shown neurotropic capacity *in vitro* models (9–11), as well as CNS inflammation and demyelination (9). Therefore, psychiatric symptoms are theoretically feasible, but it is unclear whether they come from a direct insult of the nervous system, the psychosocial distress related to the infection, or both.

Preclinical data confirm that COVID-19 is associated with neurological and neuropsychiatric symptoms (12–15). A case series from Wuhan found that 36% of inpatients showed neurological symptoms, mainly dizziness and headache. Some of them also presented with cerebrovascular disease in the course of their illness (16). In a detailed clinical report by Paterson et al. (12), they describe para- and post-infectious encephalitis such as ADEM and transverse myelitis. Taquet et al. (15) recently examined the estimated incidence of any neurological or psychiatric diagnosis 6 months after a first COVID-19 diagnosis, finding an overall percentage of 33% and up to 46.42% on those previously admitted to an intensive care unit. The risk of affective and anxiety disorders increased compared to the control group and the risk of psychosis.

Another important nosology is Post-Acute COVID-19 syndrome, defined as persistent symptoms and delayed or long-term complications beyond 4 weeks from onset. It has been described that in the long term, the symptoms of depression and anxiety are related not so much to the severity of the medical condition as to the appearance of physical sequelae (17). Post-Acute COVID-19 syndrome courses with fatigue, dyspnea, hair loss, attention deficit, and depression. In up to 60% of patients, the most common symptom is fatigue (18), and 30% may develop depressive symptoms. There is also evidence that coronavirus has long-term repercussions on cognition. Hampshire et al. (19) describe how individuals who have survived COVID-19 respond worse on cognitive tasks than would be expected for their age and academic level.

### Psychiatry Liaison During the Pandemic

Consultation-liaison psychiatry, also known as psychosomatic medicine, is a subspecialty of psychiatry that focuses on the care of patients with comorbid psychiatric and general medical conditions at the request of the medical or surgical treating physician. During the first wave of the pandemic, many centers considered clinical psychologists and psychiatrists non-essential personnel and were discouraged from entering isolation wards of COVID-19 patients (20). For this reason, many psychiatric services reduced their workload (21–23), although there are singular examples of Psychiatry Liaison increased referrals (24).

Psychiatrists had diverse duties during the peak of the pandemic. For example, drugs used experimentally to treat the infection (such as protease inhibitors) interacted with psychotropics through the P450 cytochrome system. As a result, they could prolong the QT interval (25–27), so medication adjustments were necessary. Patients with previous psychiatric history treated with psychotropic drugs were at a high risk of developing adverse effects or abrupt changes in drug levels in plasma through pharmacokinetic interactions, so they required close follow-up during hospitalization. Corticoids used to reduce inflammation carry the risk of severe psychiatric side effects: they can cause affective and psychotic symptoms and increase the risk of relapse in those already diagnosed with a psychiatric disorder (28).

We aimed to describe the profile of SARS-CoV-2 positive patients in which a psychiatry consultation was required in our hospital. We also described our psychiatric interventions during the peak of the pandemic.

## Materials and Methods

### Study Design

This is a cross-sectional descriptive study. This study was carried out within the Department of Psychiatry of Cruces University Hospital (Basque Country, Spain), a hospital that comprises 900 inpatient beds and covers 550,000 people. Data from each psychiatric consultation within our consultation-liaison service were consecutively obtained during 1 month from March 17 (4 days after the state of emergency was declared in Spain) to April 17, 2020. A total of 721 hospitalizations of SARS-CoV-2 positive patients took place during this time frame.

### Participants and Sources of Information

In the present study, we used a non-probability sampling method. All COVID-19 inpatients that were consecutively referred to our psychiatry liaison service were selected for analysis. Sociodemographic variables such as age, gender, and psychiatric history according to the International Classification of Diseases (ICD-10) were extracted from patient history. We considered past psychiatric history as any psychiatric diagnosis of the ICD-10. The severity of pneumonia was assessed using the CURB 65 scale, an instrument used to determine the severity of pulmonary infections that also serve as a predictor of prognosis in the coronavirus infection (29, 30). We also collected the referrals' characteristics, such as date, sources (medical specialties), and primary reasons for consultation. Finally, we took other variables regarding intervention and outcome: psychopharmacological intervention, number of visits, and destination at discharge.

The Service of Preventive medicine provided general data on the number of hospitalized COVID-19 infections.

### Type of Intervention

We carried out two basic types of intervention: face-to-face and telephonic interviews. Following CDC recommendations, whenever possible, telematic communication was preferred to limit healthcare workers' exposure to the virus. We stated that telephonic interviews would initially manage referrals whose objective was crisis intervention or anxiety management. In general, those patients presenting with psychotic symptoms, severe behavioral disturbances, and suicide ideation were evaluated face-to-face from the beginning. We also provided familiar crisis intervention if needed. Medication interactions between COVID-19 treatment and concomitant psychotropic drugs were in all cases assessed and switched to safer options if indicated by clinical guidelines.

#### Telephonic Crisis Intervention

A crisis can be defined as a period of psychological disequilibrium triggered by a hazardous event. We followed the guide of the Crisis Intervention by Telephone book by Lester (31). The use of telephone interviews is controversial; some authors stated that its use could even question the profession of psychiatry (32). However, according to the extensive research by Lester, telematic counseling may even be more effective in some situations, such as acute anxiety or those disorganized patients that cannot handle face-to-face intervention. Telephone interviews have unique features: they potentiate patient control and anonymity, facilitating self-revelation and openness; it is an accessible and immediate way of communication and can even promote positive transference (31). A metanalysis published by Hubley et al. revealed that patients and providers were both satisfied with telepsychiatry. It was comparable to face-to-face in terms of reliability of clinical assessments and treatment outcomes (33).

#### Face-to-Face Intervention

Our team was equipped with protective clothing, surgical gloves, face shields, and FFP2 face masks during all face-to-face interventions. In addition, we kept a minimum of 2 m between the staff and the patient and avoided all physical contact with patients and objects inside the room.

### Statistical Methods

Descriptive statistics for the study were performed. Categorical data were reported as frequencies and percentages, while continuous data were presented as median and standard deviation. Statistical analysis was carried out using IBM^®^ SPSS^®^ Statistics version 25.0 (IBM GmbH, Ehningen, Germany).

### Ethics

The Cruces University Hospital Ethics Committee approved this study as part of an ongoing database within our psychiatry department.

## Results

### Patients

We received a total of 43 SARS-CoV-2-positive referrals, of which *n* = 16 (37.2%) were men and *n* = 27 (62.8%) were women. In 9.3% of patients, the intervention was requested for a family intervention. The median age was 61 (SD 14) years old. The median age for men was 62.2 (SD 17.6) and 60.5 (SD 11.5) for women.

A complete list of clinical data is shown in Table 1. The majority of patients (58.1%) presented past psychiatric history at the moment of the referral, and most of them (44.2%) had two or more coexisting organic illnesses.

**Table 1:** Clinical characteristics of COVID-19 psychiatry liaison referrals.

Sex	Male: 16 (37.2%)
	Female: 27 (62.8%)
Age (median)	61 (SD 14)
Psychiatric diagnosis	No psychiatric history: 18 (41.9%)
	F.05 Delirium: 3 (7%)
	F.07 Organic personality disorders … 1 (2.3%)
	F.10 Alcohol 1 (2.3%)
	F.20 Schizophrenia: 1 (2.3%)
	F.22 Delusional disorder: 1 (2.3%)
	F.25 Schizoaffective disorder: 2 (4.7%)
	F.28 Other psychotic disorders: 1 (2.3%)
	F.31 Affective bipolar disorder 2 (4.7%)
	F.32 Depressive episode: 3 (7.0%)
	F.33 Recurrent depression: 3 (7.0%)
	F.41 Other anxiety disorders: 2 (4.7%)
	F.43 Adjustment disorder: 3 (7.0%)
	F. 69 Borderline personality: 1 (2.3%)
	F.73 Severe cognitive impairment: 1 (2.3%)
Comorbilities	No coexisting disease: 9 (20.9%)
	Hypertension: 4 (9.3%)
	Diabetes: 1 (2.3%)
	Coronary disease: 1 (2.3%)
	COPD: 2 (4.7%)
	Cancer: 1 (2.3%)
	Chronic renal disease: 1 (2.3%)
	Two or more: 19 (44.2%)
	Others: 5 (11.6%
Treatment	Antivirals: 36 (83.7%)
	Antibiotics: 28 (65.1%)
	Corticoids: 20 (46.5%)
	Immunoglobulins: 1 (2.3%)
	Hydroxychloroquine: 33 (76.7%)
	Mechanical ventilation: 10 (23.3%)


Regarding COVID infection severity at hospital admission, most patients had a CURB 65 score of 0 (37.2%), a score of 1 in 20.9%, 2 in 11.6%, and 3 in 9.3%. A total of 20.9% of patients did not have the score calculated at the moment of hospitalization. Frequent treatments comprised antivirals, hydroxychloroquine, and corticoids (Table 1). In 23.3%, mechanical ventilation was needed.

### Referral Features

**Figure 1:**
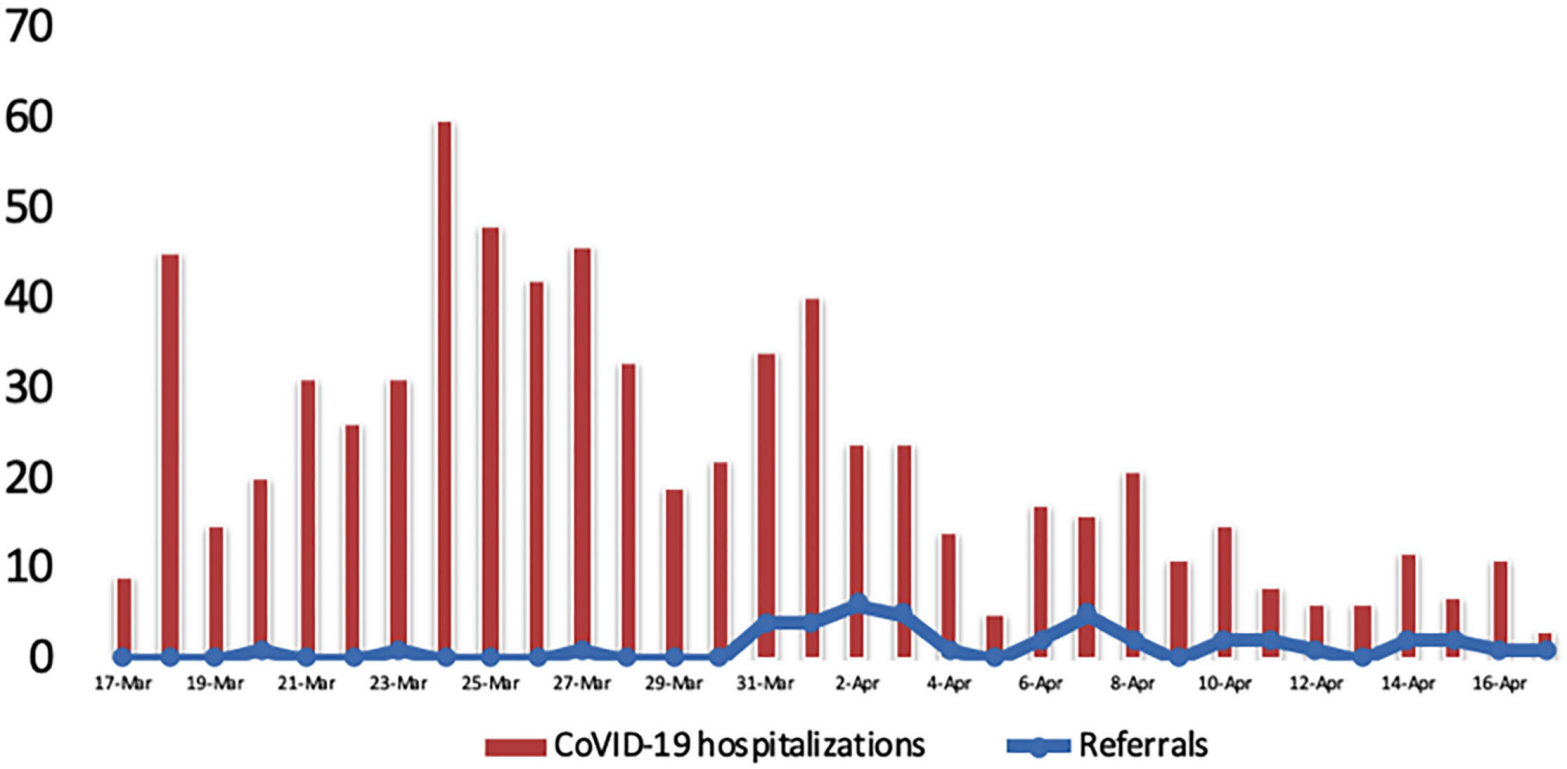
Total daily hospitalized SARS-CoV-2 infections are depicted in vertical red charts. The blue line represents the evolution of daily psychiatry liaison referrals.

A total of 721 patients were admitted to our hospital as inpatients due to COVID infection during the study period. Of them, 43 needed a psychiatric evaluation (5.6%) (Figure 1). The services requesting a psychiatric consultation and the reasons for it are depicted in Table 2. The most frequent source of referrals was the Infectious Disease Department, reaching up to 37.2% of the requests. The most frequent reason was patient anxiety (25.6%) followed by depressive mood (18.6%) and adjustment of medication (18.6%).

**Table 2:** Psychiatric diagnosis after evaluation of the referrals.

ICD-10 diagnosis	No psychiatric diagnosis: 1 (2.3%)
	F.05 Delirium: 8 (18.6%)
	F.20 Schizophrenia: 1 (2.3%)
	F.22 Delusional disorder: 1 (2.3%)
	F.25 Schizoaffective disorder: 2 (4.7%)
	F.28 Other psychotic disorders: 1 (2.3%)
	F.30 Manic episode: 1 (2.3%)
	F.31 Affective bipolar disorder 2 (4.7%)
	F.32 Depressive episode: 2 (4.7%)
	F.41 Other anxiety disorders: 1 (2.3%)
	F.43 Adjustment disorder: 5 (11.6%)
	F. 69 Borderline personality: 1 (2.3%)
	F.73 Severe cognitive impairment: 1 (2.3%)
	Z code: 14 (32.5%)


### Psychiatric Intervention and Outcome

Patients received a median of 3.7 visits/calls during hospitalization. We visited men a median of 4.1 (SD 1.7) times and women a median of 3.5 (SD 2.5) times, but these differences did not reach statistical significance.

In 58.1% of the referrals, the intervention took place by telephone. The rest needed a face-to-face approach. Regarding psychotherapeutic interventions, 67.4% received psychological counseling or psychotherapy support services. Psychological counseling was more frequent in telephone interventions than in face-to-face assessments (84 vs. 44.4%), reaching statistical significance (*p* < 0.05).

Over the total amount of patients, antipsychotics were prescribed in 34.9% and benzodiazepines in 20.9%. No other drug classes such as SSRI or mood stabilizers were initiated in any individual. There was a need for treatment switch in 20.9% of patients to avoid the risk of pharmacokinetic interactions. In all cases, safer options were administered until the end of the COVID-19 course of treatment following guideline recommendations.

The most frequent diagnosis (32.5%) was *Z-code (Factors influencing health status and contact with health services*) categorized in the ICD-10. Delirium not induced by alcohol or other drugs (F.05) was diagnosed in 18.6% of patients. Finally, there was one manic episode that was categorized as treatment-related and one depressive episode with psychotic symptoms that required psychiatric hospitalization. The complete list of psychiatric diagnoses at discharge is represented in Table 3.

**Table 3:** Referral features (*n* = 43).

Source (Specialty)	Intensive care unit: 5 (11.6%)
	Internal medicine: 6 (14%)
	Respiratory medicine: 11 (25.6%)
	Infectious disease: 16 (37.2%)
	Gynecology and obstetrics: 2 (4.7%)
	Emergency medicine: 2 (4.7%)
	Cardiology: 1 (2.3%)
Reason	Anxiety: 11 (25.6%)
	Low mood: 8 (18.6%)
	Family crisis: 4 (9.3%)
	Delirium: 2 (4.7%)
	Medication adjustment: 8 (18.6%)
	Follow-up of psychiatric patients: 2 (4.7%)
	Suicidal ideation: 1 (2.3%)

Regarding outcomes, hospital-to-home discharge occurred in 27 (62.8%) of patients. A total of 11 (25.6%) remained hospitalized at the moment of the analysis, and 3 (7%) were transferred to another medical hospital. One individual (2.3%) was sent to an inpatient psychiatry facility, and 1 (2.3%) of the 43 individuals died.

## Discussion

In our study, we analyzed the features of the psychiatric referrals during the first wave of the pandemic. In total, 5.6% of the COVID-19 patients admitted to our hospital needed a psychiatric evaluation at the request of the referent doctor. This number is lower than other studies in which 25% of coronavirus-infected patients required a psychiatric assessment (34).

We observed that the referral rate changed over time: it remained low while the hospitalization curve trended upward and increased after the peak of the first wave (Image 1). We hypothesize that during the rising phase of the curve, when little was known about the virus and protocols were in constant change, all referrals were kept to a minimum to avoid medical staff exposure and save protection equipment. On March 31, we implemented a phone-based psychological support program, explaining the increase in referrals. Another explanation may be a general reduction of activity, seen not only in psychiatry but also in other disciplines (35, 36), although we did not specifically evaluate this in our study. For example, Butler et al. found a 40% reduction of liaison psychiatry consultations during the first wave (23). Other studies report a marked decrease of referrals, specifically during March 2020, which returned to normal afterward (37). These data contrast with studies during the second wave in which the number of liaison psychiatry consultations increased by 18.8% (22).

In our sample, we found a high proportion of acute anxiety and low mood symptoms, in line with previous studies that analyzed the prevalence of depression and anxiety in COVID-19 patients (38–40). Complete isolation during treatment, fear of the unknown, or the lack of emotional support over the hospitalization may explain this finding. Still, the infection already increases the risk of anxiety or affective disorders (13, 41). In addition to this, previous research has established “sickness behavior” (42), a pattern of adaptive behavioral changes that occur in both animals and humans in response to infection or inflammatory processes. It consists of low mood, anxiety, and social isolation, which we also observed in our COVID-19 referrals.

Delirium is a common symptom of COVID-19 disease, ranging from 25 to 33% in previous studies (43, 44). It constituted 18.6% of the diagnosis made in our referrals. Although not reported, most delirious patients presented with florid psychotic symptoms and behavioral disturbances that were, in some cases, a diagnostic challenge. However, other characteristic features such as fluctuating course and attention impairment were almost always present. Delirium is usually multifactorial: the old age of our patients, with a median age of 61 years old, polypharmacy, and the infection itself may contribute to its development.

In our sample, the medication used to treat the infection comprised mainly antivirals and hydroxychloroquine, which were used experimentally during the first wave of the pandemic. Ritonavir is a well-known CYP3A4 inhibitor, and therefore in 20.3% of our total referrals, a change in medication was necessary to avoid interactions. If we consider only those patients with a prior psychiatric history, this percentage goes up to 37.5%. This confirms previous research reporting an elevated risk of pharmacokinetic interactions in psychiatric patients (28), a fact that acquires particular relevance in a SARS-CoV-2 infection setting. In addition, we report a case of first episode of mania presumably attributed to steroid therapy that was effectively addressed with Olanzapine. We did not identify any first psychotic episodes. However, it has been proved that the risk of a psychotic disorder is augmented during the SARS-CoV-2 illness, and there are case reports of new-onset psychosis after infection (15, 45–47).

In our study, we also describe the interventions by our team. Although some centers decided to evaluate all patients face-to-face (34), our protocol stated telephonic intervention would be preferred when possible. Still, 42.9% of patients had to be interviewed face-to-face to carry out an optimal psychopathological exploration, a situation that in all cases required us to wear Personal Protective Equipment (PPE). The median visit per patient was 3.7, but one patient needed up to 17 interventions due to the severity of his symptoms and the longer-than-average length of COVID-19 hospitalizations. In other cases, face-to-face interviews were unavoidable due to the often scarce and confusing information obtained by phone calls, leading to doubts in the diagnosis.

## Strengths and Limitations

We registered all the referrals to our psychiatric liaison service in a snowball fashion; therefore, there is a risk of bias, and the results cannot be extrapolated to the general population. Also, the small sample limits the statistical power of potential comparations within groups. Nevertheless, the main strength of our study is that it represents a comprehensive picture of the role of a psychiatry service during the first wave of the pandemic, with a specific focus on the interventions by liaison subspecialty. However, as we did not use psychometric scales, it is also difficult to compare results.

## Conclusions

For psychiatrists working in liaison psychiatry, the novel COVID-19 was a challenge in which the course of action with hospitalized patients had to be reformulated. It forced psychiatrists to change their previous intervention settings and working strategies. More research is needed not only to obtain a complete picture of the psychiatric symptoms of the coronavirus disease but also to understand the different patterns of psychiatry liaison care during the pandemic.

## Data Availability Statement

The raw data supporting the conclusions of this article will be made available by the authors, without undue reservation.

## Ethics Statement

The present study was approved by the Cruces University Hospital Ethics Committee as part of an ongoing observational study in our psychiatry department. This study aims to describe all the patients treated in our unit, both inpatients and outpatients. Due to the exceptional circumstances regarding COVID-19 isolation policies, written informed consent could not be systematically signed and we obtained verbal consent instead. In those patients whose capability was impaired, a legal representative was informed and gave consent. Patient anonymity was in all cases preserved and we analyzed only aggregated data.

## Author Contributions

ML-A, JG-B, SF-S, JC-G, and ÁP-R collected patient data. AA-A collected epidemiological data of total hospitalized infections. ML-A and AA-A analyzed the data. ML-A, JC-G, and MS-H wrote the manuscript. All authors approved the final work.

## Conflict of Interest

The authors declare that the research was conducted in the absence of any commercial or financial relationships that could be construed as a potential conflict of interest.

## Publisher's Note

All claims expressed in this article are solely those of the authors and do not necessarily represent those of their affiliated organizations, or those of the publisher, the editors and the reviewers. Any product that may be evaluated in this article, or claim that may be made by its manufacturer, is not guaranteed or endorsed by the publisher.
